# Datasets on factors influencing the urban environmental quality of intra-urban motor parks across density areas of Lagos metropolis

**DOI:** 10.1016/j.dib.2018.06.116

**Published:** 2018-07-03

**Authors:** Olayinka O. Agunloye, Olabisi O. Ajakaiye, Adedotun O. Akinola, Hilary I. Okagbue, Adedeji O. Afolabi

**Affiliations:** a Department of Urban and Regional Planning, University of Lagos, Lagos, Nigeria; b Department of Urban and Regional Planning, Yaba College of Technology, Lagos, Nigeria; c Department of Architecture, Covenant University, Canaanland, Ota, Nigeria; d Department of Mathematics, Covenant University, Canaanland, Ota, Nigeria; e Department of Building Technology, Covenant University, Nigeria

**Keywords:** Likert scale, Survey analytics, Motor parks, Lagos, Statistics, Environment

## Abstract

This survey data examined the factors influencing commuters’ perception of environmental quality in the selected intra-urban motor parks of Ibeju Lekki, Ifako Ijaiye and Ikeja local government areas, Lagos State, Nigeria. A survey of 376 commuters was carried out. The purposive sampling technique was used for the survey while the sampling procedure evolved from the identification of the study area to the administration of questionnaire with commuters in the motor parks. Data were analyzed using descriptive (likert scale outputs) and inferential statistical techniques (factor analysis for data reduction and categorization). The datasets can be considered in the transport and environmental policies of Lagos State and Nigeria with a view to engendering a conducive environment in the intra-urban motor parks of Lagos State, Nigeria.

**Specification Table**

**Table:** 

Subject area	Environmental Science
More specific subject area	Transportation Management
Type of data	Tables
How data was acquired	Field Survey through questionnaire
Data format	Raw and analyzed
Experimental factors	Simple percentages and commuter perception index (CPI) were used as analytical tool of the generated data. Factor analysis was used in determining the factors influencing environmental quality in intra-motor parks. Likert scale also ranked factors using the Sum of weighted values (SWV).
Experimental features	The key method used in data collection - structured questionnaire designed in Likert scale, the questionnaire was designed in such a way that it helped to collate basic information from the respondents. A population size of seventy five thousand, thirty two (75,032) was selected, and a total sample size of 376 respondents was used in data generation, with questionnaire distributed to commuters. Variables pertaining to the above listed targets were identified and incorporated into questionnaires as the primary source of data. The data was collated and analyzed using mean item score ranking, percentages, descriptive statistics and inferential statistics.
Data source location	Ibeju Lekki, Ikeja and Ifako-Ijaiye Local Government Areas,Lagos State, Nigeria
Data accessibility	All collected data are in this data article

**Value of the data**

• The data can be used for evolving transportation and environmental policy for Lagos State, Nigeria.
• The data could be used in location and infrastructure planning of motor parks for Lagos State, Nigeria.
• The survey can be adopted for other high density cities in Nigeria such as Abuja, Kano, Kaduna, Ibadan, Enugu, Calabar, Warri, Benin City, Port-Harcourt and so on.
• The data could be used as basis of comparison of environmental quality of intra-urban motor parks across other density areas of Lagos metropolis and Nigeria at large.
• The questionnaire for this survey can be adopted and adapted in other subject areas.
• The data can be used by the physical planning authority (government) and private developers as a framework in addressing the subject of environmental quality in the location, design and planning of other urban motor parks and similar infrastructures taking into consideration the Commuter׳s perception.

## 1 Data

The data describes collated responses solicited from commuters on their take on the factors influencing commuters’ perception of environmental quality in the selected intra-urban motor parks of Ibeju Lekki, Ifako Ijaiye and Ikeja local government areas, Lagos State, Nigeria. A total of 400 questionnaires was distributed and 376 (94%) were retrieved for analysis. Non response were excluded from the analysis. Data collected through the research instrument was analyzed and provided study information. Previous studies on the subject can be seen in [1], [2], [3], [4], [5], [6], [7], [8], [9], [10]. The following methods of analysis were employed: Descriptive statistics (univariate analysis) using mean, frequency, percentages and proportions. The five point likert scale was also used. The various responses were analyzed using the Statistical Package for Social Sciences (SPSS Version 21).

### 1.1 Socio-demographic characteristics of commuters

The socio-economic characteristics of the commuters explore the socio-demographic differences in the factors influencing commuters’ perception of environmental quality. These include: gender (Table 1), age (Table 2), education status (Table 3), employment status (Table 4), monthly income (Table 5), marital status (Table 6) and household size (Table 7).

**Table 1:** Gender of respondents.

**Gender**	**Ibeju-Lekki**		**Ifako**		**Ikeja**		**Total**	
	Freq	%	Freq	%	Freq	%	Freq	%
Male	22	62.9	107	58.2	92	58.6	221	58.8
Female	13	37.1	77	41.8	65	41.4	155	41.2
**Total**	35	100.0	184	100.0	157	100.0	**376**	**100**

**Table 2:** Age of respondents.

**Age of respondent**	**Ibeju-Lekki**		**Ifako**		**Ikeja**		**Total**	
	Freq	%	Freq	%	Freq	%	Freq	%
Below 18 Years	2	5.7	21	11.4	10	6.4	33	8.7
18–40 Years	19	54.3	113	61.4	97	61.8	229	60.9
40–60 Years	11	31.4	40	21.7	44	28.0	95	25.3
Above 60 Years	3	8.6	10	5.4	6	3.8	19	5.1
**Total**	**35**	**100.0**	**184**	**100.0**	**157**	**100.0**	**376**	**100**

**Table 3:** Education status of respondents.

**Education status of respondent**	**Ibeju-Lekki**		**Ifako**		**Ikeja**		**Total**	
	Freq	%	Freq	%	Freq	%	Freq	%
No formal education	–	–	15	8.2	23	14.6	38	10.1
Primary education	2	5.7	9	4.9	14	8.9	25	6.7
Secondary education	16	45.7	45	24.5	41	26.1	102	27.1
Tertiary (first degree)	17	48.6	83	45.1	62	39.5	162	43.1
Post graduate	–	–	32	17.4	17	10.8	49	13.0
**Total**	**35**	**100.0**	**184**	**100.0**	**157**	**100.0**	**376**	**100**

**Table 4:** Employment status of respondents.

**Employment status of respondent**	**Ibeju-Lekki**		**Ifako**		**Ikeja**		**Total**	
	Freq	%	Freq	%	Freq	%	Freq	%
Yes	25	71.4	113	61.4	110	70.0	248	66
No	10	28.6	71	38.5	47	29.9	128	34
**Total**	**35**	**100.0**	**184**	**100.0**	**157**	**100.0**	**376**	**100**

**Table 5:** Monthly income of respondents.

**Monthly income of respondents**	**Ibeju-Lekki**		**Ifako**		**Ikeja**		**Total**	
	Freq	%	Freq	%	Freq	%	Freq	%
Below N18,000	9	25.7	46	25.0	44	28.0	99	26.3
N18,000–N36,000	11	31.4	72	39.1	58	36.9	141	37.5
N36,000–N54,000	5	14.3	26	14.1	10	6.4	41	10.9
N54,000–N72,000	7	20.0	13	7.1	18	11.5	38	10.1
N72,000–N90,000	3	8.6	17	9.2	16	10.2	36	9.6
Above N90,000	–	–	10	5.4	11	7.0	21	5.6
**Total**	**35**	**100.0**	**184**	**100.0**	**157**	**100.0**	**376**	**100**

**Table 6:** Marital status of respondents.

**Marital status of respondent**	**Ibeju-Lekki**		**Ifako**		**Ikeja**		**Total**	
	Freq	%	Freq	%	Freq	%	Freq	%
Single	12	34.3	103	56.0	63	40.1	178	47.3
Married	18	51.4	59	32.1	78	49.7	155	41.2
Divorced	1	2.9	10	5.4	6	3.8	17	4.5
Widowed	1	2.9	7	3.8	4	2.5	12	3.2
Separated	3	8.6	5	2.7	6	3.8	14	3.8
**Total**	**35**	**100.0**	**184**	**100.0**	**157**	**100.0**	**376**	**100**

**Table 7:** Household size of respondents.

**Household size of respondent**	**Ibeju-Lekki**		**Ifako**		**Ikeja**		**Total**	
	Freq	%	Freq	%	Freq	%	Freq	%
1 Person	5	14.3	8	4.3	4	2.5	17	4.5
2 Persons	4	11.4	20	10.9	8	5.1	32	8.5
3 Persons	5	14.3	24	13.0	24	15.3	53	14.1
4 Persons	7	20.0	49	26.6	40	25.5	96	25.5
5 Persons	11	31.4	37	20.1	30	19.1	78	20.7
6 Persons	1	2.9	30	16.3	26	16.6	57	15.2
7 Persons	1	2.9	11	6.0	14	8.9	26	6.9
8 Persons	1	2.9	3	1.6	6	3.8	10	2.7
9 Persons	–	–	2	1.1	1	.6	3	0.8
10 Persons	–	–	8	4.3	3	1.9	11	2.9
11 Persons	–	–	–	–	1	0.6	1	0.3
**Total**	**35**	**100.0**	**184**	**100.0**	**157**	**100.0**	**376**	**100**

In summary, data revealed that young adults (18–40 years), literates (graduates of tertiary institutions), employed, underpaid and married persons, were most affected by the environmental quality of the intra-urban motor parks across the three density areas in Lagos metropolis.

## 2 Experimental design, materials and methods

A survey of intra-urban motor parks of Ibeju Lekki, Ifako Ijaiye and Ikeja local government areas, Lagos State, Nigeria. The target population was chosen because the area is densely populated and often experience heavy vehicular movements. Secondly, they contain several motor parks that link to the other parts of the state. Studies [11], [12], [13], [14], [15], [16], [17], [18], [19], [20], [21], [22], [23], [24], [25], [26], [27], [28], [29], [30] have used similar statistical methodologies in analyzing their survey data. Simple percentages and commuter perception index (CPI) were used as analytical tool of the generated data.

Section A of the questionnaire was used to extract data on the socio-demographic characteristics of the commuters (respondents). Section B of the questionnaire had questions on “factors influencing environmental quality”. The data were extracted using 5-Likert type scale, where 1 is for “Strongly disagree”; 2 is for “Disagree”; 3 represents “Moderately agree”; 4 is for “Agree”; and 5 represents “Strongly disagree. The questionnaire can be assessed as Supplementary Data 1 while the raw data for the three local Government area considered can be assessed as Supplementary Data 2.

Factor analysis was used in determining the factors influencing environmental quality in intra-motor parks. Likert scale also ranked factors using the sum of weighted values (SWV). The factors influencing environmental quality as summarized using the CPI and SWV as shown in Table 8. It can be seen that the factors were arranged in decreasing order of the commuter perception index. Some statistical test was carried out to test the reliability of the data for factor analysis. The results are presented in Table 9. It can be seen that the KMO value is 0. 913 with Bartlett׳s test significance of 0.000. This indicates that the data is suitable for factor analysis. The tests further indicate that the correlation matrix is not an identity matrix. Further indices such as Cronbach׳s Alpha can be included. Communalties of variables were obtained as presented in Table 10. The principal component analysis was used to collapse 33 variables. The variable with the lowest communality was lighting (58.5%) while the highest communality was odor (88.7%). Total variance explained using the principal component analysis as extraction method was shown in Table 11. It can be seen that all factors that are with Eigenvalues above 1 were extracted and represented under the column extraction sums of square loadings. The results revealed 7 unconfirmed factors and also suggested that there was a cumulative total of 71.61% with variances of 3.09% and 5.94% at and after extraction; which was confirmed after rotational extraction. The rotated component matrix of factors influencing commuters’ perception of environmental quality was presented in Table 12. The result revealed the structure of variables that were studied and used in the reduction into four factors. These factors are physical, economic and recreational and educational factors. The component transformation matrix of factors influencing commuters’ perception of environmental quality was presented in Table 13. As with the others, principal component analysis was used as the extraction method and varimax with Kaiser Normalization was used as the rotation method.

**Table 8:** Factors influencing environmental quality.

**S/N**	**Factors**	**Opinion**					**SWV**	**CPI**
		**1**	**2**	**3**	**4**	**5**		
1	Distance to work	23	79	150	88	100	1483	3.94
2	Availability of Market	9	55	120	160	33	1282	3.41
3	Lighting	14	49	145	134	34	1253	3.33
4	Accessibility to road network	12	70	141	105	48	1235	3.28
5	Accessibility to Transport	15	83	128	100	50	1215	3.23
6	Public water supply	21	69	121	137	28	1210	3.21
7	Toilet Condition	29	67	122	129	29	1190	3.16
8	Building Condition	22	51	197	63	43	1182	3.14
9	Security of Passengers	16	86	147	86	41	1178	3.13
10	State of the toilet facilities	27	74	106	117	42	1171	3.11
11	Accessibility to economic opportunity	19	99	109	125	24	1164	3.09
12	Cost of Living	19	69	161	113	14	1162	3.09
13	Drainages	50	66	97	127	36	1161	3.09
14	Building Density	8	77	205	53	33	1154	3.06
15	Cost of Food	21	65	171	108	11	1151	3.06
16	Cost of Rent	31	63	161	102	19	1143	3.04
17	Information Boards	42	81	115	102	36	1137	3.02
18	Security of Cars	20	109	124	90	33	1135	3.02
19	Borehole	43	84	116	94	39	1130	3.0
20	Traffic Density	16	111	128	98	23	1129	3.0
21	Road Condition	32	85	136	104	19	1121	2.98
22	Litterbins	42	97	102	100	35	1117	2.97
23	Car Park	25	98	141	93	19	1111	2.95
24	Nearness to health facility	25	104	140	77	30	1111	2.95
25	Availability of Shops	11	41	148	155	21	1107	2.94
26	Aesthetics	35	88	143	88	22	1102	2.93
27	Signages	34	95	139	91	17	1090	2.89
28	Cleanliness	58	74	126	96	22	1078	2.86
29	Shelter	58	74	126	96	22	1078	2.86
30	Footpath/Pedestrian walkway	27	117	135	89	8	1062	2.82
31	Picnic Benches	60	107	121	68	30	1059	2.82
32	Landscaping	42	101	135	80	18	1059	2.82
33	Physically Challenged Accessibility	28	138	137	55	18	1025	2.76
34	Privacy	45	117	116	80	18	1037	2.75
35	Social Interaction among neighbors	48	101	145	72	10	1023	2.72
36	Sitting Platform	61	85	111	93	16	1016	2.70
37	Nearness to Secondary School	32	116	138	62	28	1004	2.67
38	Open Spaces	31	114	137	80	14	990	2.63
39	Air Pollution	83	99	86	92	16	987	2.60
40	Presence of Hazard	67	120	112	55	22	973	2.59
41	Odor	97	88	81	95	15	971	2.58
42	Dust and Silt	85	98	102	71	20	971	2.58
43	Well Water	63	130	105	59	19	969	2.57
44	Privacy Level	81	112	98	59	26	965	2.57
45	Nearness to Primary School	44	118	137	57	20	962	2.55
46	Noise Level	97	103	74	79	23	956	2.54
47	Water Fountain	83	135	94	46	18	955	2.53
48	Flora	82	124	96	62	12	926	2.46
49	Children Play Facility	83	123	97	55	17	870	2.31
50	Fuana	94	139	95	41	7	856	2.27

Strongly disagree (1), Disagree (2), Moderately agree (3), Agree (4), Strongly disagree (5).

**Table 9:** KMO and Bartlett׳s Tests of factors influencing environmental quality.

Kaiser-Meyer-Olkin Measure of Sampling Adequacy.		0.913
Bartlett׳s Test of Sphericity:	Approx. Chi-Square	9062.745
	Degree of freedom	528
	Significant level	0.000

**Table 10:** Communalties of factors influencing environmental quality.

Variables	Initial	Extraction
Distance to work	1.000	0.598
Accessibility to transport	1.000	0.742
Accessibility to road network	1.000	0.791
Traffic density	1.000	0.625
Privacy	1.000	0.612
Accessibility to economic opportunity	1.000	0.628
Availability of shops	1.000	0.655
Public water supply	1.000	0.720
Litter bins	1.000	0.728
Information boards	1.000	0.667
Children׳s play facility	1.000	0.738
Nearness to primary school	1.000	0.786
Nearness to secondary school	1.000	0.861
Nearness to health facility	1.000	0.742
Social interaction among neighbours	1.000	0.592
Cost of food	1.000	0.816
Cost of living	1.000	0.782
Cost of rent	1.000	0.823
Aesthetics	1.000	0.696
Picnic benches	1.000	0.734
Seating platform	1.000	0.712
Drainages	1.000	0.707
Availability of market	1.000	0.614
Lighting	1.000	0.585
Presence of hazard	1.000	0.657
Security of cars	1.000	0.723
Security of passengers	1.000	0.599
State of the toilet facilities	1.000	0.659
Air pollution level	1.000	0.794
Dust and silt	1.000	0.837
Odour	1.000	0.887
Noise level	1.000	0.827
Privacy level	1.000	0.696

Extraction method: principal component analysis.

**Table 11:** Total variance explained of the factors influencing environmental quality.

Component	Initial Eigenvalues			Extraction sums of squared loadings			Rotation sums of squared loadings		
	Total	% of Variance	Cumulative %	Total	% of Variance	Cumulative %	Total	% of Variance	Cumulative %
1	11.885	36.015	36.015	11.885	36.015	36.015	4.879	14.784	14.784
2	3.856	11.686	47.700	3.856	11.686	47.700	4.450	13.485	28.269
3	2.347	7.113	54.813	2.347	7.113	54.813	3.334	10.105	38.374
4	1.776	5.383	60.196	1.776	5.383	60.196	3.246	9.836	48.210
5	1.492	4.520	64.716	1.492	4.520	64.716	3.000	9.091	57.301
6	1.254	3.801	68.517	1.254	3.801	68.517	2.764	8.374	65.675
7	1.021	3.094	71.611	1.021	3.094	71.611	1.959	5.936	71.611
8	0.872	2.642	74.253						
9	0.711	2.155	76.408						
10	0.685	2.076	78.485						
11	0.641	1.942	80.426						
12	0.589	1.784	82.211						
13	0.511	1.548	83.759						
14	0.505	1.530	85.289						
15	0.466	1.411	86.700						
16	0.426	1.291	87.991						
17	0.411	1.245	89.236						
18	0.369	1.118	90.353						
19	0.326	0.988	91.341						
20	0.321	0.972	92.313						
21	0.302	0.914	93.227						
22	0.285	0.862	94.089						
23	0.253	0.768	94.857						
24	0.246	0.744	95.601						
25	0.218	0.661	96.262						
26	0.205	0.621	96.883						
27	0.188	0.570	97.453						
28	0.174	0.526	97.980						
29	0.167	0.505	98.484						
30	0.151	0.458	98.943						
31	0.141	0.429	99.371						
32	0.126	0.380	99.752						
33	0.082	0.248	100.000						

Extraction method: principal component analysis.

**Table 12:** Rotated component matrix of factors influencing commuters’ perception of environmental quality.

	Component						
	1	2	3	4	5	6	7
Odour	0.911						
Dust and silt	0.893						
Noise level	0.886						
Air pollution level	0.865						
Presence of hazard	0.682						
Privacy level	0.637		0.330				
Accessibility to road network		0.802					
Accessibility to transport		0.786					
Traffic density		0.699					
Distance to work		0.598		0.388			
Security of cars		0.548				0.523	
Accessibility to economic opportunity		0.521	0.360				0.364
Privacy		0.502	0.393				
Security of passengers		0.468			0.362	0.425	
Childrens׳ play facility			0.733		0.362		
Picnic benches			0.731				
Seating platform		0.359	0.622				
Information boards		0.391	0.554				
Aesthetics		0.325	0.532	0.467			
Litter bins		0.430	0.483				0.440
Cost of food				0.869			
Cost of rent				0.860			
Cost of living				0.818			
Nearness to secondary school					0.875		
Nearness to primary school					0.836		
Nearness to health facility					0.769		
Social interaction among neighbours	0.384	0.306		0.301	0.407		
Lighting						0.648	
State of the toilet facilities						0.600	
Availability of market				0.386		0.569	
Drainages		0.361	0.431			0.519	
Availability of shops							0.729
Public water supply							0.700

Extraction method: principal component analysis.

Rotation method: Varimax with Kaiser normalization.

Rotation converged in 7 iterations.

**Table 13:** Component transformation matrix of factors influencing commuters’ perception of environmental quality.

Component	1	2	3	4	5	6	7
1	0.415	0.528	0.434	0.300	0.298	0.335	0.264
2	− 0.787	0.052	− 0.051	0.434	0.390	0.094	0.162
3	0.392	− 0.463	− 0.114	0.642	0.195	− 0.407	0.064
4	0.157	− 0.032	− 0.262	− 0.414	0.834	− 0.023	− 0.193
5	0.162	− 0.193	− 0.570	0.167	− 0.105	0.756	0.038
6	0.039	0.609	− 0.407	0.296	− 0.102	− 0.211	− 0.565
7	0.053	0.308	− 0.486	− 0.149	− 0.063	− 0.312	0.737

Extraction method: principal component analysis.

Rotation method: Varimax with Kaiser normalization.
