# Supplementary material for: Datasets on factors influencing the urban environmental quality of intra-urban motor parks across density areas of Lagos metropolis
Source: Data Brief. 2018 Jul 3;19:2109–18. doi: 10.1016/j.dib.2018.06.116 (PMC6141441; doi:10.1016/j.dib.2018.06.116)
Supplement: Supplementary file 1 — Transparency document. [file mmc1.pdf]

COVENANT UNIVERSITY

COLLEGE OF SCIENCE AND TECHNOLOGY

DEPARTMENT OF MATHEMATICS

CANAANLAND, KM 10, IDIROKO ROAD
P.M.B 1023, OTA, OGUN STATE, NIGERIA
www.covenantuniversity.edu.ng, mat.covenantuniversity.edu.ng

EXTERNAL MEMO

To: Editor, Data in Brief
From: Corresponding Author
Date: 29th May, 2018
Subject: **Conflict of Interest**

I thereby declare the absence of any conflict of interest among the authors.

The authors have read the final draft and unanimously agreed that the paper be sent for review.

Hilary I. Okagbue
Department of Mathematics, College of Science and Technology, Covenant University, Ota.
Google Scholar: *Hilary Izuchukwu Okagbue*
Research Gate: *Hilary Okagbue*
Live DNA: 234.14453
Scopus ID: 56438006100
ORCID: 0000-0002-3779-9763
