# Supplementary material for: Datasets on factors influencing the urban environmental quality of intra-urban motor parks across density areas of Lagos metropolis
Source: Data Brief. 2018 Jul 3;19:2109–18. doi: 10.1016/j.dib.2018.06.116 (PMC6141441; doi:10.1016/j.dib.2018.06.116)
Supplement: Supplementary file 3 — Supplementary Data 2. [file mmc3.docx]

**UNIVERSITY OF LAGOS**

**SCHOOL OF POST GRADUATE STUDIES**

**DEPARTMENT OF URBAN AND REGIONAL PLANNING**

Dear respondent,

Your response will greatly help to proffer solution to the **Environmental Quality of Intra-Urban Motor Parks in Selected Density Areas of Lagos Metropolis**. Please note that data captured will be treated with utmost confidentiality and will be used solely for the purpose of this research work.

Thank you.

**Section A (Socio-economic characteristic of commuters)**

1. What is your gender? a. Male b. Female
2. How old are you? a. below 18 yrs b. 18 – 40 c. 40 – 60 d. above 60
3. What is your present education status? a. no formal education b. primary school c. Secondary school d. tertiary 1^st^ degree e. post graduate
4. Are you employed: a. yes b. no
5. How much do you earn monthly? a. below N18,000 b. N18,000 – N36,000 c. N36,000 – N54,000 d. N54,000 – N72,000 e. N72,000 – 90,000 f. Above N90,000
6. Which is your marital status: a. Single b. Married c. divorced d. widowed e. separated
7. What is your household size? ………………………………………………..

**Section B (Environmental Condition of the motor park)**

1. Rate the environmental condition of the following in the motor park

| Condition  Variables | Very bad (1) | Bad  (2) | Fair  (3) | Good  (4) | Very good  (5) |
| --- | --- | --- | --- | --- | --- |
| Toilets |  |  |  |  |  |
| Drainage |  |  |  |  |  |
| Litter bins |  |  |  |  |  |
| Pedestrian walkways |  |  |  |  |  |
| General sanitation |  |  |  |  |  |
| Open space |  |  |  |  |  |
| security |  |  |  |  |  |
| Shops |  |  |  |  |  |
| Market |  |  |  |  |  |
| Aesthetics |  |  |  |  |  |
| Sign/information board |  |  |  |  |  |
| Shelter/waiting area |  |  |  |  |  |
| Roads |  |  |  |  |  |
| Lightening |  |  |  |  |  |
| Benches |  |  |  |  |  |

**Section C (Level of Satisfaction of commuters concerning facilities in the motor park)**

1. Rate your level of satisfaction of the following facilities in the motor park.

| Level of satisfaction  Facilities | Very dissatisfied (1) | Dissatisfied (2) | Moderately satisfied (3) | Satisfied (4) | Very satisfied  (5) |
| --- | --- | --- | --- | --- | --- |
| Roads |  |  |  |  |  |
| Car parks |  |  |  |  |  |
| Footpath/pedestrian walkway |  |  |  |  |  |
| Signs |  |  |  |  |  |
| Disabled access |  |  |  |  |  |
| Landscaping |  |  |  |  |  |
| Pond/river/lake/ water fountain |  |  |  |  |  |
| Flora |  |  |  |  |  |
| Fauna |  |  |  |  |  |
| Open Space |  |  |  |  |  |
| Shelter |  |  |  |  |  |
| Toilets |  |  |  |  |  |
| Liter – bins |  |  |  |  |  |
| Information board |  |  |  |  |  |
| Children’s play facility |  |  |  |  |  |
| Picnic benches |  |  |  |  |  |
| Seating platform |  |  |  |  |  |
| Drainage |  |  |  |  |  |
| Market shops |  |  |  |  |  |
| Lighting |  |  |  |  |  |

**Section D (Factors influencing environmental quality of the motor park)**

1. On a scale of 1 – 5, rate how much you agree that the following variables influence environmental quality in the motor park

| Level of agreement/  Factor | Strongly disagree (1) | Disagree (2) | Moderately agree (3) | Agree (4) | Strongly disagree (5) |
| --- | --- | --- | --- | --- | --- |
| Roads |  |  |  |  |  |
| Car parks |  |  |  |  |  |
| Footpath/pedestrian walkway |  |  |  |  |  |
| Signs |  |  |  |  |  |
| Disabled access |  |  |  |  |  |
| Landscaping |  |  |  |  |  |
| Pond/river/lake/ water fountain |  |  |  |  |  |
| Flora |  |  |  |  |  |
| Fauna |  |  |  |  |  |
| Open Space |  |  |  |  |  |
| Cleanliness |  |  |  |  |  |
| Shelter |  |  |  |  |  |
| Toilets |  |  |  |  |  |
| Well water |  |  |  |  |  |
| Borehole |  |  |  |  |  |
| Building condition |  |  |  |  |  |
| Building density |  |  |  |  |  |
| Distance to work |  |  |  |  |  |
| Accessibility to transport network |  |  |  |  |  |
| Accessibility to road network |  |  |  |  |  |
| Traffic density |  |  |  |  |  |
| Privacy in neighbourhood |  |  |  |  |  |
| Accessibility to economic opportunities |  |  |  |  |  |
| Availability of shops |  |  |  |  |  |
| Public tap water supply |  |  |  |  |  |
| Liter – bins |  |  |  |  |  |
| Information board |  |  |  |  |  |
| Children’s play facility |  |  |  |  |  |
| Nearness to primary school |  |  |  |  |  |
| Nearness to secondary school |  |  |  |  |  |
| Nearness to health facility |  |  |  |  |  |
| Social interaction among neighbours |  |  |  |  |  |
| Cost of food |  |  |  |  |  |
| Cost of living |  |  |  |  |  |
| Cost of rent |  |  |  |  |  |
| Aesthetics |  |  |  |  |  |
| Picnic benches |  |  |  |  |  |
| Seating platform |  |  |  |  |  |
| Drainage |  |  |  |  |  |
| Availability of market |  |  |  |  |  |
| Lighting |  |  |  |  |  |
| Presence of hazard |  |  |  |  |  |
| Security – cars in motor park |  |  |  |  |  |
| Security – personnel safety in motor park |  |  |  |  |  |
| Hygiene (toilet) |  |  |  |  |  |
| Smoke |  |  |  |  |  |
| Dust/silt |  |  |  |  |  |
| Odour |  |  |  |  |  |
| Noise |  |  |  |  |  |
| Privacy |  |  |  |  |  |

1. Suggest ways/strategies of improving the environmental quality of the motor park ………………………………………………………………………………………….
